# Protective effects of education on the cognitive decline in a mental rotation task using real models: a pilot study with middle and older aged adults

**DOI:** 10.1007/s00426-022-01719-2

**Published:** 2022-07-30

**Authors:** Martina Rahe, Claudia Quaiser-Pohl

**Affiliations:** University of Koblenz-Landau, Universitaetsstrasse 1, 56070 Koblenz, Germany

## Abstract

Mental rotation is the ability to rotate objects in one’s mind. Large age-related decreases in accuracy and processing time are often found in studies using paper-and-pencil or computerized mental rotation tests. For older participants, these tests are often too difficult. In the present study, real models consisting of cube figures were used to assess the mental rotation performance of middle and older aged adults. It should be investigated whether these tests were comparable to paper-and-pencil or chronometric tests and if very old participants were able to solve them. Eighty-four participants (49 females) between 40 and 90 years took part and were divided into middle (40–68 years) and older aged (69–90 years) and groups with higher (with college degree) and lower education (without college degree). For accuracy, main effects of gender and age group as well as interactions of age group and education were found. Younger participants outperformed older ones only in the group with lower education. For processing time, a main effect of age group as well as an interaction of age group and education was found. The age-related cognitive decline in the higher educated group was moderate, while a large effect appeared for the group without college degree. Age and gender effects of our new test with real objects were comparable to paper–pencil and computerized tests. Furthermore, a protective effect of education on the cognitive decline in mental rotation performance is discussed.

## Introduction

Mental rotation is defined as the fast and accurate rotation of two- or three-dimensional objects in one’s mind (Shepard & Metzler, 1971). The ability can be measured with chronometric (Shepard & Metzler, 1971) or psychometric (Vandenberg & Kuse, 1978) mental rotation tests or using real models (Hawes et al., 2015). Chronometric mental rotation tests usually consist of two cube figures or alternative stimuli presented on a computer display (Jansen-Osmann & Heil, 2007). Participants have to decide as fast and as accurately as possible whether both figures are identical or not. Identical figures are rotated in depth or in picture plane and non-identical figures (distractors) can be mirrored to the main figure or structurally different (Boone & Hegarty, 2017). In most chronometric tests, there is no time limit but the reaction time can be measured. Psychometric mental rotation tests often consist of the same objects used in chronometric tests but are presented as paper–pencil tests. Usually participants have to compare one target figure to four comparison figures in a given time of, for example, 3 min for 12 items (Peters et al., 1995). In most chronometric and psychometric mental rotation tests, three-dimensional objects are presented on a two-dimensional paper or screen. This can be avoided using tangible models as rotational material (Felix et al., 2011; Hawes et al., 2015). Hawes et al. (2015) used a test with one target cube figure model that had to be compared to three other cube figures, one identical figure, one mirror figure, and one structurally distinct figure. Participants (aged 4–8) had to point to the identical figure. Hawes et al. stated (2016) that psychometric mental rotation tests can be too difficult for young children because of the 2D presentation of 3D stimuli, the comparison of five objects, and the time constraint. The same arguments could apply for elderly adults. Like young children, older participants could have difficulties solving paper–pencil or computerized mental rotation tests because their cognitive abilities have declined with age (e.g., Maitland et al., 2000). Jansen and Heil (2009) assumed a floor effect for 60–70 year old participants solving an average of 4–5 out of 24 items in the psychometric test version. Therefore, we used a mental rotation test with real models to compare performance and gender differences in middle aged and elderly adults. Older senior (aged over 60 years) were tested because we wanted to investigate whether these participants would be able to solve the test and no floor effects would appear. To analyze if gender differences in adulthood in the newly developed test would be comparable to those found in paper–pencil and chronometric mental rotation tests, middle-aged adults (40–60 years) were also asked to participate. Because we assumed the test was too easy for younger adults, we did not include college students.

Regarding the differentiation of two-dimensional presentations of two- or three-dimensional objects, gender differences were found for the 3D but not for the 2D objects, the 2D task was easier than the 3D task, and the interaction of gender and task type was significant as well (Roberts & Bell, 2003). Moreover, different brain activation was found for the task type: In men, the 2D task was associated with greater left parietal activation and in women, with greater right parietal activation. On the other hand, in the 3D task, both men and women showed more right parietal activation. Regarding the comparability of mental rotation tests with real three-dimensional objects and paper–pencil tests with two-dimensional illustrations of three-dimensional objects, Felix et al. (2011) found effects of gender and stimulus type but no interactions of both. Men outperformed women and both sexes solved more items in the test with real objects than in the paper–pencil test. These results emphasize that gender differences are independent on the stimulus type and that the test with real objects is easier to solve.

### Gender differences in mental rotation performance

Gender differences in mental rotation performance favoring males appear in adulthood (Voyer et al., 1995). This effect of gender was found for different mental rotation tests (Voyer & Jansen, 2016; Weigelt & Memmert, 2021). The extent of the gender differences depends on various factors, e.g., the test version (psychometric/chronometric) (Voyer et al., 1995), the time limit (Voyer, 2011), or the objects used as rotational material (Jansen-Osmann & Heil, 2007; Rahe et al., 2021).

Gender differences are larger in psychometric than in chronometric tests (Voyer et al., 1995). In psychometric mental rotation tests, men outperformed women in younger, middle, and older adulthood (Jansen & Heil, 2009) with gender differences decreasing with age. Herman and Bruce (1983) also found gender differences in younger and older adults that remained comparable in both age groups. In chronometric tests, gender differences were found for accuracy but not for reaction time and mental rotation speed in 60–71 year old participants (Jansen & Kaltner, 2014).

### Mental rotation across adulthood

Performances in cognitive abilities usually decrease from younger to older adulthood (Maitland et al., 2000; Salthouse, 2019). For spatial abilities, a meta-analysis found a large age-related decrease for accuracy and an even larger effect for the response time (Techentin et al., 2014). Comparable results with moderate negative correlations between age and accuracy and negative large correlations between age and attempted items as a measure for processing speed were found in older adults solving a psychometric mental rotation test (Rahe et al., 2018). Better performances in younger adults were found in other psychometric mental rotation tests as well (Herman & Bruce, 1983; Iachini et al., 2019; Jansen & Heil, 2009). In chronometric mental rotation tests, men outperformed women in accuracy (Jansen & Heil, 2009), reaction time, and mental rotation speed (Jacewicz & Hartley, 1987).

To the best of our knowledge, no study has investigated the mental rotation performance using real models in elderly adults. Real models were used as training material to improve the performance of elderly participants (Meneghetti et al., 2018). After training with the models, participants performed better in mental rotation tasks and in some transfer tasks, e.g., a fluid intelligence task.

For the assessment of mental rotation abilities in elderly adults and for comparison studies of younger or middle aged and older adults, real models could be useful. If computerized tests are used, younger participants could have advantages because they are more used to computers, visual displays on a screen, and the operation of the keyboard. For paper–pencil tests as well as for computerized tests, participants have to imagine the objects on the two-dimensional screen or paper as three-dimensional figures. This transformation process can be avoided using real models and therefore, the mental rotation process can be measured more isolated. Moreover, the time limit in most paper–pencil tests could also disadvantage elderly people because they aim to give the correct answer more than younger people do (Sharps & Gollin, 1987). Furthermore, there are usually four comparison figures in a paper–pencil test item. This could be more difficult for older compared to younger or middle aged adults. Therefore, other processes can interfere with the mental rotation process and disturb the comparability of the results of younger and older participants. A mental rotation test with two real comparison objects and no time limit seems to be better suited for elderly participants and therefore for a comparison of two age groups.

### Influence of educational differences on cognitive aging

The cognitive decline due to aging does not proceed in the same way for all people (Clouston et al., 2020; Rowe & Kahn, 1997; Whalley et al., 2004). The model of a cognitive reserve is suggested when it comes to dementia and to differences of brain damages and its clinical outcomes (Stern, 2002, 2009). Cognitive reserve is defined as “individual differences in how people process task [that] allow some to cope better than others with brain pathology” (Stern, 2009, p. 2016). Besides higher childhood intelligence or more complex occupation (Finkel et al., 2009), higher education seems to be an important protective factor to attenuate negative outcomes of brain damages or dementia (Stern, 2009). However, relationships of education and aging are complex and dependent of the task (Ardila et al., 2000) and sample (Gow et al., 2012) and there are studies that found no positive effects of education in normal cognitive aging (Proust-Lima et al., 2008; Van Dijk et al., 2008). In spatial tasks, age and gender were the strongest predictors of spatial performance but only small effects of education and no interactions of age and education were found (Herrera-Guzman et al., 2004). Veldema and Jansen (2019) tested seniors over 80 years in a paper–pencil mental rotation test with two dimensional pictures of animals as rotational material. No gender differences were found in these age group, but participants with a college degree significantly outperformed those without a degree. In their meta-analysis of spatial performance, Techentin et al. (2014) found a moderator effect of education on the effect of aging. The effect size of age was significantly larger if the younger sample was more educated than the older sample compared to studies without differences in education across age groups.

### Goal of the study

First, the study aims to show that gender, age, and educational differences in mental rotation performance measured with paper-and-pencil or computerized tests are comparable to our test with real models. Furthermore, we want to investigate whether an age related decline in mental rotation performance is dependent of education. We assume that participants with a higher education do not show such a steep age-related decline in performance as participants without a higher education.

#### Hypotheses

In line with previous studies investigating mental-rotation performance in paper–pencil or chronometric tests, we predicted better performance for men than for women (Voyer et al., 1995) and for younger participants than for older one (Techentin et al., 2014). Moreover, participants with a college degree should outperform those without a degree (Clouston et al., 2020). Furthermore, it should be investigated whether age related declines in performance are dependent on gender or education.

## Methods

### Participants

In this study, 84 participants (49 females and 35 males) between 40 and 90 years (*M* = 67.94, SD = 13.78) took part. For moderator analyses, median splits were calculated for age (up to 68 and above 69) and for education (with and without college degree). *χ*^2^—tests revealed no deviation from an even distribution between gender and age group, gender and educational group, and age group and educational group. In the younger age group were 22 women and 17 men between 40 and 68 years of age (*M* = 54.79, SD = 6.89) and in the older age group were 27 women and 18 men between 70 and 90 years of age (*M* = 79.33, SD = 5.60). Participants were recruited in church groups, choirs, and in their private and professional environment.

### Materials

#### Mental rotations task

**Fig. 1:**
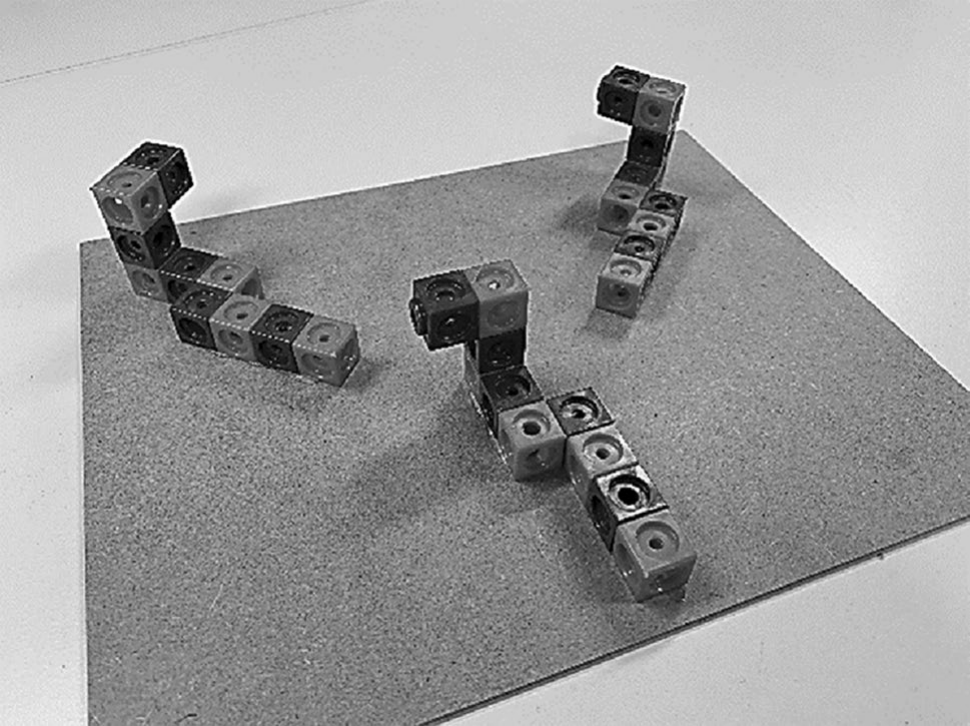
Example item of the mental rotation test

For the mental rotation test, real models were constructed consisting of three plastic cube figures on a small board for each item (Fig. 1). Each cube figure was constructed of ten small snap cubes (alternating red and blue, height of one cube: 1.7 cm, board size: 20 × 30 cm) according to the paper-and-pencil mental-rotation test of Vandenberg and Kuse (1978). For each item, a main figure was placed in the front of a board and two comparison figures were placed in the rear of the board, right and left of the main figure. One of the comparison figures was identical but rotated to the main figure, the second comparison figure was a mirrored version of the main figure. This configuration was chosen because the original psychometric mental rotation test with five objects could be too difficult for very old participants. When only two objects are presented and participants have to decide whether these two were identical or mirror reversed, accuracy and reaction time is usually calculated only for items with identical objects (for an overview see Jost & Jansen, 2020). The identical comparison figure was rotated at 45° or 135° to the right or to the left to the main figure.

### Procedure

All participants were tested alone in a quiet room. Participants were seated at a table opposite of the experimenter. The first model was used to explain the task. The board was placed in front of the participant with the main figure facing the participant and the comparison figures facing the experimenter. The experimenter explained that one of the comparison figures was identical to the main figure while the other was a mirrored version. The participants were instructed to point towards the identical figure as quickly and as accurately as possible. As an indicator for the processing time, the experimenter started a timer when she placed the board in front of the participant and stopped it when the participant pointed towards one of the objects. During the practice item, the participants were allowed to take their time to get familiar with the task and to ask any questions they had. After the practice item, participants had to solve nine test items; two of the identical objects were rotated at 45° to the main figure and seven were rotated at 135°. A total number of ten items was chosen so the testing would not be too long for the very old. For the same reasons, only two rotational angles were chosen. For processing time, the mean time (in sec) per item was calculated and for accuracy, the number of correctly solved items was measured.

All participants gave their written consent for participation. The experiment was conducted according to the ethical guidelines of the Helsinki declaration. Ethical approval for this study was not required in accordance with the conditions outlined by the German Research Foundation where research that carries no additional risk beyond daily activities does not require Research Ethics Board Approval.

### Statistical analysis

First, a paired *t* test was calculated to analyze if participants took more time for the larger angle of rotation than for the smaller one. The dependent variable was the processing time and the within-subject factor were both angles of rotation (45° and 135°). To test our hypotheses, two regression analyses with backwards method were conducted to analyze predictors of the mental rotation performance (processing time and number of correct items). Moreover, two univariate analyses of variance with the dependent variables processing time and number of correct items and the independent variables gender, age group, and college degree were calculated.

## Results

For processing time, the paired t-test revealed a main effect of the within-subjects factor rotational angle, *t*(83) = 2.376, *p* = 0.020, *d* = 0.259. Participants answered faster when the rotational angle was 45° (*M* = 12.59, SD = 10.67) compared to the items with a rotational angle of 135° (*M* = 14.85, SD = 11.95). Furthermore, we found a negative correlation between processing time and number of correct answers, *r*(84) = − 0.256, *p* = 0.019.

A regression analysis with the dependent variable processing time and the predictors gender, age, college degree, and the interaction of age and college degree was calculated. Age, *β* = 0.598, *p* < 0.001, and age × degree, *β* = − 0.192, *p* = 0.029, were significant predictors. Both predictors explained 38% of the variance of the processing time, *F*(2,81) = 26.538, *p* < 0.001, corr*R*^2^ = 0.381. For the number of correct answers, a regression analysis with the same predictors was calculated. Age, *β* = − 0.538, *p* < 0.001, gender, *β* = − 0.343, *p* < 0.001, college degree, *β* = − 1.124, *p* = 0.024, and age × degree, *β* = 1.225, *p* = 0.012, were significant predictors. All predictors explained 27% of the variance of the number of correct answers, *F*(4,79) = 8.520, *p* < 0.001, corr*R*^2^ = 0.266.

**Fig. 2:**
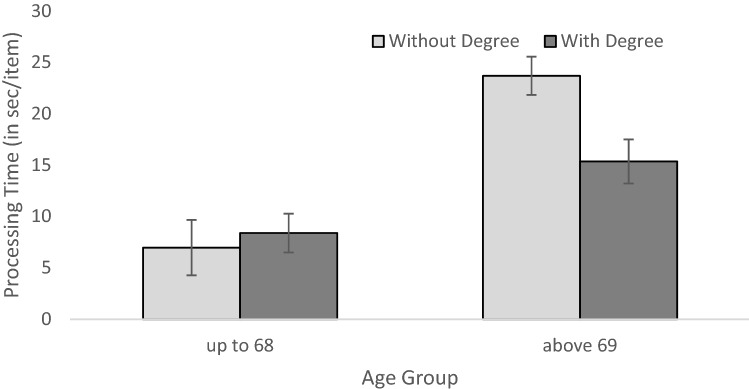
Processing time per item as a function of age group and educational group. Error bars indicated SE

The univariate analysis of variance with processing time per item as dependent variable and gender, age group, and college degree as independent variables showed a main effect of age group, *F*(1,76) = 29.682, *p* < 0.001, η_p_^2^ = 0.281, as well as a significant interaction of age group and college degree, *F*(1,76) = 5.030, *p* = 0.028, η_p_^2^ = 0.062 (Fig. 2). Young participants solved the test faster (*M* = 8.02, SD = 6.56) than older participants (*M* = 19.82, SD = 11.38). In the younger age group, no significant effect of college degree was found while in the older age group, participants with a degree processed the task significantly faster than participants without a degree, *F*(1,76) = 8.619, *p* = 0.004, η_p_^2^ = 0.102. Concerning age differences, a medium effect size of age was found in the group with degree, *F*(1,76) = 5.938, *p* = 0.017, η_p_^2^ = 0.072, and a large effect size in the group without degree, *F*(1,76) = 26.061, *p* < 0.001, η_p_^2^ = 0.2^55^. No other main effects or two- or three way interactions were significant.

**Fig. 3:**
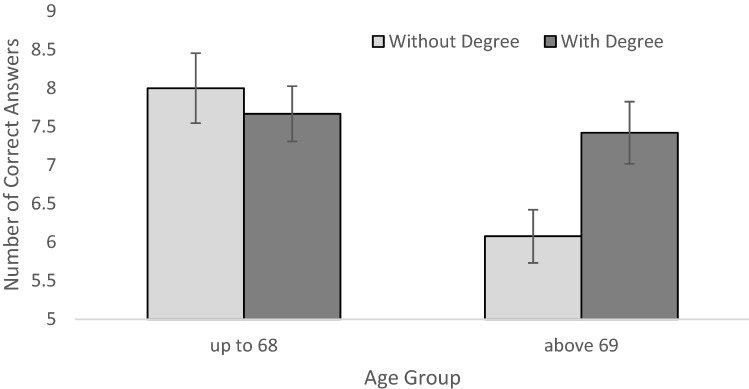
Number of correctly solved items as a function of age group and educational group. Error bars indicated SE

A univariate analysis of variance with number of correct items as dependent variable and gender, age group, and college degree as independent variables revealed main effects of gender, *F*(1,76) = 9.249, *p* = 0.003, η_p_^2^ = 0.108, and age group, *F*(1,76) = 5.983, *p* = 0.017, η_p_^2^ = 0.073, and a significant interaction of age group and college degree, *F*(1,76) = 4.856, *p* = 0.031, η_p_^2^ = 0.060 (Fig. 3). Males (*M* = 7.94, SD = 1.24) outperformed females (*M* = 6.63, SD = 2.10) and younger participants (*M* = 7.79, SD = 1.84) solved more items correctly than older participants (*M* = 6.64, SD = 1.79) did. The interaction indicates an effect of college degree only for the older age group, *F*(1,76) = 5.858, *p* = 0.018, η_p_^2^ = 0.072, but not for the younger group, *F*(1,76) = 0.676, *p* = 0.413, η_p_^2^ = 0.009. Regarding age differences, younger participants outperformed older ones only in the group without degree, *F*(1,76) = 9.524, *p* = 0.003, η_p_^2^ = 0.111, but not in the college degree group, *F*(1,76) = 0.034, *p* = 0.854, η_p_^2^ < 0.001. No other main effects or two- or three way interactions were significant.

## Discussion

In our mental rotation test with real models, we found age differences with a moderate effect size for accuracy and a large effect size for processing time. Main effects of education did not appear and gender differences in favor of men were found for accuracy but not for processing time. Significant interactions with moderate effect sizes of age group and education group appeared for accuracy and processing time.

### Gender differences in mental rotation

Gender differences did not appear for processing time, which is in line with Jansen-Osmann and Heil (2007) for chronometric tests. Gender differences in accuracy were also comparable in effect size with participants between 40 and 70 years in a study of Jansen and Heil (2009). In the present study with real models, women did not take more time to solve the tasks but men solved the tasks more accurately. A meta-analysis of gender differences in spatial tasks revealed larger effects in favor of men for psychometric than for chronometric mental rotation tests (Voyer et al., 1995). Our items had more comparison figures than the usual chronometric test but less than a psychometric test. Absent time pressure and instructions to answer as quickly and as accurately as possible are more comparable to the chronometric test. In the present study, we found gender differences in accuracy, which are comparable with Voyer et al.’s (1995) effect size for psychometric mental rotation tests.

### Mental rotation across adulthood

Age group differences are in line with the meta-analysis of Techentin et al. (2014). They found larger age effects for processing time than for accuracy. The cognitive decline seems to be stronger in the processing speed of cognitive tasks than in the accuracy (Jacewicz & Hartley, 1987). Cerella et al. (1981) also found comparable accuracies for older and younger participants but a slower rotational process in older people. More recent studies found the same pattern: The age related decline was stronger for processing time than for accuracy (Rahe et al., 2018; Zhao et al., 2020). Therefore, our mental rotation task with real models seems to be comparable to psychometric tests referring to effects of age.

Experimental conditions emphasizing the speed or the accuracy (or both) of a chronometric mental rotation tasks, resulted in longer response times for elderly compared to younger participants in the accuracy but not in the speed condition (Sharps & Gollin, 1987). If speed is important, elderly people seem to be able to process the task as fast as younger one. However, if accuracy is stressed out, older participants take more time to make sure their answer is correct. Surprisingly, accuracy did not differ in both conditions (Sharps & Gollin, 1987). In the speed/accuracy condition, which is comparable to the instruction in the present study, accuracy was comparable across age groups while elderly people needed significantly more time for the task. Hence, older participants seem to take more time than younger adults do to make sure their answer is correct. Therefore, a test without a time limit can judge older participants’ abilities more fairly than a paper–pencil test does.

Many studies (Cerella et al., 1981; Puglisi and Morrell, 1986; Zhao et al., 2020) comparing younger and elderly adults examine college students as younger participants. That can result in different structures of education, gender, and life style. The advantage of our sample is the equal distribution of women and men as well as people with and without college degree in both age groups. Furthermore, we did not examine college students who are still in a qualification process and therefore, are more used to cognitive demands and testing.

### Influence of educational differences on cognitive aging

No significant main effects of education were found for accuracy or processing time. Reasons for that could be the high concreteness and tangibility of the task: in relation to a psychometric or chronometric test, the objects were real and participants did not have to be able to envision two-dimensional figures on a screen or sheet as three-dimensional objects. That could have made the spatial cognitive task less difficult, which could be in favor of less educated people.

Furthermore, we found interaction effects of education and age for accuracy and processing speed. This is in line with a study of Alley et al. (2007) who found a slower rate of cognitive decline for participants that are more educated. However, they did not test their participants’ cognitive abilities but used the Telephone Interview for Cognitive Status (TICS), which is rather a measure of self-evaluated cognitive abilities or mental status. Our results of the protective effect of education on the decline in mental rotation performance is in contrast to a study of Van Dijk et al. (2008) who found no protective effects of education on normal cognitive aging for a 6 years span in healthy individuals between 49 and 81 years. Anstey and Christensen (2000) also found evidence that education was no protective factor on the cognitive decline in fluid intelligence but in measures of mental status, memory, and crystallized abilities. For mental rotation performance, strong correlations of participants’ confidence in their abilities with their actual performance were found (Cooke-Simpson & Voyer, 2007; Estes & Felker, 2012). It can be assumed that higher educated elderly people with a better mental status (Anstey & Christensen, 2000) and higher self-evaluated cognitive abilities (Alley et al., 2007) have a higher confidence in their own abilities. This higher confidence could then lead to a better performance of older adults with a higher education compared to older adults without a college degree. Comparable results of younger participants between 40 and 68 years with and without a higher education could be due to the real models. Because of their professional occupation, less educated participants could be more familiar with concrete objects than people with a college degree are and therefore have an advantage with that part of the task.

Veldema and Jansen (2019) found differences between participants with and without a college degree in people above 80 years of age. In the present study, the regression analyses showed a significant interaction of age and education as well and the ANOVAs confirmed the interaction of age and education. The effect of a higher education was significant in the older age group but not in the younger group. These results emphasize the protective effect of a higher education on the cognitive decline in spatial abilities. To avoid the pronounced cognitive decline in the less educated elderly, lifelong learning and the occupation with challenging activities especially in retirement age should be promoted. Besides better motor ability (Jansen & Kaltner, 2014) or a specific training (Meneghetti et al., 2018), that could be a promising approach to mitigate the cognitive decline.

### The mental rotation task with real models

Our mental rotation test with real objects seems to be less difficult to solve than paper–pencil tests. With a mean score of six items out of nine in participants over 69 years without a college degree, the task seems to be feasible even for the oldest. For accuracy in the psychometric mental rotation test, Jansen and Heil (2009) assumed a possible floor effect in the performance of their oldest participants. Therefore, the psychometric mental rotation test could be less suited than a test with real models to measure spatial abilities and to detect gender differences in the elderly. The application of the test for younger children could also be considered as mental rotation tests with cube figures are often considered as too difficult for children in elementary school (Jansen et al., 2013).

### Limitations and future research

The number of items was rather small and the capturing of the processing time was a rough measurement without a shield. Moreover, the measurement of education was rather superficial. For young adults (e.g., college students), the test with real models could be too easy to solve and therefore produce ceiling effects in the accuracy. In future research, very old participants could be tested with our test with real objects and the paper–pencil test. It could then be investigated whether floor effects in the paper–pencil test do not appear in the test with real object. Furthermore, a possible training effect of the real objects on the paper–pencil test would be interesting to analyze. Solving the test with real objects first could help older participants to visualize the 3D objects and enhance their accuracy in a paper–pencil test. Moreover, in further studies, participants should be allowed to touch and rotate the objects manually. On this basis, a new motor training test could be developed.

### Conclusion

This study gives a first hint that a higher education could have a protective effect on the cognitive decline in a mental rotation task with real models. Furthermore, effects of gender and age on the performance in the mental rotation task with real models seem to be comparable to other mental rotation tests. It can be assumed that the task is less difficult because of the concreteness of the rotational objects. Therefore, the test could be used to measure spatial abilities in middle and older adulthood and avoid possible floor effects in the oldest.

## Data Availability

The datasets generated during and/or analyzed during the current study are available from the corresponding author on reasonable request.
